# Dead or dying? Quantifying the point of no return from hydraulic failure in drought‐induced tree mortality

**DOI:** 10.1111/nph.15922

**Published:** 2019-07-08

**Authors:** William M. Hammond, Kailiang Yu, Luke A. Wilson, Rodney E. Will, William R. L. Anderegg, Henry D. Adams

**Affiliations:** ^1^ Plant Biology, Ecology and Evolution Oklahoma State University Stillwater OK 74078 USA; ^2^ School of Biological Sciences University of Utah Salt Lake City UT 84112 USA; ^3^ Department of Natural Resource Ecology and Management Oklahoma State University Stillwater OK 74078 USA

**Keywords:** climate change, drought, ecophysiology, foliar color, hydraulic failure, tree die‐off, tree mortality, tree physiology

## Abstract

- Determining physiological mechanisms and thresholds for climate‐driven tree die‐off could help improve global predictions of future terrestrial carbon sinks. We directly tested for the lethal threshold in hydraulic failure – an inability to move water due to drought‐induced xylem embolism – in a pine sapling experiment.
- In a glasshouse experiment, we exposed loblolly pine (*Pinus taeda*) saplings (*n* = 83) to drought‐induced water stress ranging from mild to lethal. Before rewatering to relieve drought stress, we measured native hydraulic conductivity and foliar color change. We monitored all measured individuals for survival or mortality.
- We found a lethal threshold at 80% loss of hydraulic conductivity – a point of hydraulic failure beyond which it is more likely trees will die, than survive, and describe mortality risk across all levels of water stress. Foliar color changes lagged behind hydraulic failure – best predicting when trees had been dead for some time, rather than when they were dying.
- Our direct measurement of native conductivity, while monitoring the same individuals for survival or mortality, quantifies a continuous probability of mortality risk from hydraulic failure. Predicting tree die‐off events and understanding the mechanism involved requires knowledge not only of when trees are dead, but when they begin dying – having passed the point of no return.

## Introduction

The Earth is undergoing rapid shifts in ecosystem structure and composition due to an unprecedented rate of warming accompanied by increased variability in precipitation driven by anthropogenic climate change (IPCC, 2014). Forests in many regions around the world have experienced elevated rates of tree mortality and episodes of widespread, regional forest die‐off (Allen *et al*., 2010). Trees provide important ecosystem services, including erosion prevention, hydrologic balance and maintaining biodiversity (Anderegg *et al*., 2013; Morillas *et al*., 2017; Hartmann *et al*., 2018a), and also dominate terrestrial carbon sequestration, contributing more to global carbon sink strength per land area than any other vegetation type (Bonan, 2008). The fate of feedbacks in carbon exchange between forests and the atmosphere under a changing climate remains one of the largest uncertainties in projecting future climate change (Friedlingstein *et al*., 2006, 2014; Friend *et al*., 2014). Despite the importance of forest die‐off, predicting when and where it will occur in response to climate remains a challenge for vegetation modeling. Development of process‐based models that simulate the physiological mechanisms of stress and mortality may be the best solution for prediction of rapid, nonlinear tree mortality events under future climate scenarios that are not analogous to current climate conditions (Allen *et al*., 2010; McDowell *et al*., 2011).

Process‐based prediction of forest mortality requires us to identify the physiological causes of tree death. Two interrelated physiological mechanisms for tree mortality from drought have been proposed: hydraulic failure and carbon starvation (McDowell *et al*., 2008). Hydraulic failure occurs during drought‐induced water stress when xylem tensions become high enough to cause air‐seeded embolism that occludes water transport beyond a threshold for survival (Sperry & Tyree, 1988; Cochard *et al*., 1992). Carbon starvation during drought is proposed to occur after stomatal closure when maintenance respiration demands exceed stored nonstructural carbohydrate (NSC) reserves via reserve exhaustion or through immobilization if reserves are inaccessible (Sala *et al*., 2010; McDowell *et al*., 2011; Sevanto *et al*., 2014).

While hydraulic failure and carbon starvation are probably highly interactive during tree mortality – and clearly are not mutually exclusive mechanisms (McDowell *et al*., 2011) – observations have demonstrated that hydraulic failure is a ubiquitous factor in tree death from drought in experiments and natural settings (Adams *et al*., 2017). In the global synthesis of Adams *et al*., hydraulic failure was > 60 PLC (per cent loss of xylem conductivity) in all cases where trees died. By contrast, reductions in NSC (compared to control groups) were observed in only 48% of cases. Resistance to hydraulic failure has also been found to predict tree species’ mortality rates during drought around the world (Anderegg *et al*., 2016; Martin‐StPaul *et al*., 2017). Carbon starvation has been observed to interact with hydraulic failure in causing tree death (O'Brien *et al*., 2014), but may only prove directly lethal on its own in the case of extreme light limitation (Sevanto *et al*., 2014; Wiley *et al*., 2017). In a shade‐mortality experiment, Wiley *et al*. found that NSC reserves in roots could be nearly exhausted (< 1% DW) at whole‐plant death, levels rarely observed in field or glasshouse studies of drought‐induced mortality (Adams *et al*., 2017). Although carbon starvation can occur in the absence of drought‐induced hydraulic failure (Wiley *et al*., 2017), it seems unlikely that any level of hydraulic failure can occur without some amount of carbon immobilization. In the present study, we quantify only hydraulic failure as a risk factor for drought‐induced tree mortality, but acknowledge a secondary effect of hydraulic failure is immobilization of some carbon stores (Sala *et al*., 2010) and, thus, measures such as PLC could be integrative of both water and carbon stress.

While previous work has often focused on comparisons between trees which are dead or probably committed to death (i.e. past the point of no return), and those which survive, we still lack an understanding of the transition space between life and death. Most importantly, we do not yet know when trees begin dying during drought stress – passing a point of no return – such that they will fail to recover and eventually die, even if drought stress is relieved. For example, hydraulic failure in studies summarized by Adams *et al*. (2017) probably occurred long after trees passed the point of no return in hydraulic function. To advance our ability to predict tree mortality, we need to pivot from describing the symptoms in trees that are already dead (i.e. past the ‘point of no return’) to quantify the risk factors that distinguish dying trees from those which will survive (Hartmann *et al*., 2018a; Martínez‐Vilalta *et al*., 2019). For hydraulic failure during prolonged drought stress, embolism continues to accrue until no xylem remains functional as tree tissues become desiccated. However, it is unlikely that complete loss of xylem conductivity is necessary to cause mortality. Trees can die when some portion of the xylem is still functional but conductivity has been reduced below a threshold sufficient for survival, such that trees will die even if water becomes available (Adams *et al*., 2017; Hartmann *et al*., 2018a).

Quantifying this threshold, the point of no return for hydraulic failure beyond which it is expected that the majority of trees in a population will die, is a current priority for understanding the mechanisms causing tree mortality (McDowell *et al*., 2011; Adams *et al*., 2017; Martínez‐Vilalta *et al*., 2019). Supply–demand hydraulic theory, based on the hydraulic constraints on water transport through plants from atmospheric demand and soil water supply, posits that exceeding some substantial level of hydraulic loss, perhaps 60 PLC, results in an increased likelihood of mortality (Sperry & Love, 2015). Model hindcasting of drought‐induced tree mortality found that a threshold in hydraulic function associated with a chronic PLC > 50 successfully predicted landscape‐scale patterns of aspen (*Populus tremuloides* Michx.) mortality (Anderegg *et al*., 2015). Results from several drought experiments were used to infer a lethal threshold near or above 60 PLC following post‐drought rewatering from either the timing of gas exchange recovery (Brodribb & Cochard, 2009; Brodribb *et al*., 2010; Urli *et al*., 2013) or thresholds in water potential as related to PLC via hydraulic vulnerability curves (Li *et al*., 2015). Although experiments, landscape‐level modeling and theory have indicated that *c. *60 PLC might be a useful predictor of drought‐induced tree mortality, hydraulic failure thresholds have not been experimentally determined with direct measurement of PLC just before relieving drought and monitoring for survival or mortality of measured individuals.

Canopy color is available from many remote sensing products and may be a useful indicator of drought‐induced tree death to facilitate the detection and monitoring of widespread drought‐induced tree die‐off (Hartmann *et al*., 2018a). Changes in foliar color have often been used in previous studies to determine whether trees are dead or alive, with a common definition of death including foliar browning (see Table S3 in Adams *et al*., 2017 for a recent review; Adams *et al*., 2009; Anderegg *et al*., 2012). However, it remains unknown whether foliar color changes (including browning) occur before, during or after the point of no return from hydraulic failure.

Here we focus on directly testing the lethal limit for hydraulic failure with a point of no return drought experiment on loblolly pine (*Pinus taeda* L.). We exposed a population of saplings (*n* = 83) to variable levels of water stress, ranging from mild to severe, before rewatering saplings once preassigned targets in predawn water potential for each treatment had been reached. Before rewatering, we directly measured PLC in stem segments from each sapling. Our objective was to characterize the risk of tree mortality associated with hydraulic failure. We hypothesized that the lethal threshold for hydraulic failure, beyond which 50% of trees would die, would be near 60 PLC, the threshold supported by theoretical, observational and inferential studies (Brodribb & Cochard, 2009; Brodribb *et al*., 2010; Anderegg *et al*., 2015; Sperry & Love, 2015). A second objective was to characterize the changes in foliar color of saplings during the experiment to determine if foliar color at rewatering was predictive of death or survival. We hypothesized that foliar color change between initial (unstressed) measurements and rewatering (relief of drought stress) for trees which died would be greater than foliar color change of trees which survived.

## Materials and Methods

We obtained 2‐yr‐old loblolly pine saplings (*n* = 83) from a local nursery (Cedar Valley Nurseries, Ada, OK, USA), grown from seeds sourced in Louisiana, USA. We transplanted saplings to 37.8‐liter pots with a soil consisting of a 2 : 1 : 1 mixture of peat moss (Peat Grower Black Bale, Berger, QC, Canada), Turface clay (MVP, Profile Products LLC, Buffalo Grove, IL, USA), and vermiculite (Ambient Minerals, Benton, AR, USA) in June 2016. We chose this mixture to create a slow, steady drying from a high initial moisture content, as this soil has a more gradual slope in the relationship between soil moisture and soil water potential than many other soils or potting mixes (Kulbaba, 2014). When transplanting, we carefully removed the nursery soil under water, such that loblolly pine saplings were planted with bare roots in our mixed soil. After transplant, all trees were kept well‐watered, received a complete fertilizer mix, (18 : 18 : 21 NPK, plus micronutrients; Miracle‐Gro, Scotts Miracle‐Gro Co. Marysville, OH, USA) supplemented with iron (Liquid Iron, Bonide, Oriskany, NY, USA) and grown for 215 d to allow acclimation in the glasshouse before we began the experiment. No trees died after transplanting. To enable trees to better acclimate to drought conditions and avoid conducting our experiment on tree saplings that had never experienced water stress, we exposed all trees, including the watered controls, to a pre‐experimental drought treatment by withholding water until stomatal closure occurred. We verified stomatal closure (> 90% reduction in stomatal conductance from well‐watered measurements) in a random subsample of the population (*n* = 36), as confirmed by measurement with an LI‐6400 infrared gas analyzer (Li‐Cor Biosciences, Lincoln, NE, USA) and a mean (SD) predawn water potential of −2.3 ± 0.25 MPa. No tree died as a result of this acclimation drought. Following this pre‐experimental drought, which lasted 43 d, trees were again well‐watered from 35 d before the experiment began. In January 2017, at the start of the drought experiment, mean (SD) sapling height was 2.02 ± 0.12 m, and mean stem diameter at the root collar (soil level) was 3.01 ± 0.24 cm. We conducted a branch census before and after the experiment and estimated that < 10% of shoot biomass was harvested for measurements during the study.

To identify rewatering targets representing a range of hydraulic failure, we constructed a vulnerability curve using six additional saplings, acquired at the same time as the 83 in our study, potted and grown in the same manner as experimental trees. First, we excised 18 branches (three each from the six individual saplings), from the most recent year's growth, 0.5–0.75 m in length, at predawn. Whole branches were recut underwater in the laboratory, allowing xylem tension to relax. Branches were then wrapped in damp paper towels, sealed in three layers of plastic bags, and shipped overnight to the University of Utah, where centrifuge measurements were conducted on stem segments. Stem segments 5–7 mm in diameter were prepared by cutting and trimming them from longer branches underwater to the desired length (*c. *10 cm). These segments were then vacuum infiltrated at 100 kPa for 1 h with degassed and filtered 20 mM KCl solution and were used to measure maximum hydraulic conductivity (Sperry *et al*., 2012). We then spun stems in a centrifuge to induce negative pressure at target values, which were −1, −2, −2.5, −3, −3.5, −4 and −5 MPa in the xylem sap. Hydraulic conductivity at each pressure stop was then determined using a conductivity apparatus as described previously (Sperry *et al*., 2012). We used a Weibull function to fit the hydraulic vulnerability curve results (Supporting Information Fig. S1).

To identify the lethal threshold of hydraulic failure, we designed our drought experiments such that trees would be rewatered at a wide range of levels of water stress. Using our centrifuge‐generated vulnerability curve as a guide (Fig. S1) and informed from a previous pilot study on a few individuals (data not included in this study), we chose specific targets at which to rewater trees and subsequently monitor for survival or mortality. We determined drought‐stress treatment groups by water potential ranges from our vulnerability curve, to ensure trees were rewatered along the entire potentially lethal drought‐stress gradient, from stomatal closure (−2.1 MPa, 20 PLC) until complete hydraulic failure (−6.0 MPa, 100 PLC). Targets for groups between these points included water potential associated with 60 (−3.3 MPa), 80 (−3.7 MPa) and 90 (−4.1 MPa) PLC. An additional group of control trees (*n* = 6) remained well watered throughout the experiment with weekly irrigation to field capacity. We measured predawn water potential of distal stems (twigs) using a Scholander‐type pressure‐bomb (model 1505‐D, PMS Instruments, Albany, OR, USA) at least weekly, and often more frequently for trees approaching rewatering targets.

At the start of the experiment, we completely withheld water from all trees except the well‐watered controls. As each tree under drought reached its assigned water potential rewatering target, we excised a branch for measurement of PLC. After sampling, each tree was rewatered to field capacity and watered weekly to monitor for survival (or death). Initial excisions of stem segments, all over 20 cm in length, were made in air, and were immediately placed in filtered (0.2 μm) 100 mM KCl solution and transported to the laboratory for measurement of hydraulic conductivity. In the laboratory, we recut stems under filtered solution by removing at least 2 cm from the cut end in two successive ≥ 1 cm cuts, allowing time for relaxation of the xylem tension, and then we immediately excised a segment of interest once tension was relaxed to prevent refilling of native embolism (Torres‐Ruiz *et al*., 2015; Wheeler et al., 2013). Immediately before measurement of hydraulic conductivity, we removed *c. *0.5 cm of bark from each end, exposing the xylem for measurement of diameter, and shaved the ends of each segment (while underwater) with a fresh razor blade. After determining conductivity, we measured segment length and perpendicular diameters of each end using digital calipers (VWR, Radnor, PA, USA) in order to correct measurements to area for specific conductivity. All measured segments collected were from the most recent year's growth, and mean diameter was 3.91 ± 0.60 mm, and mean length was 62.68 ± 10.06 mm. We also measured conductivity of the well‐watered control trees at the end of the experiment.

We measured native conductivity (*K*_s_) using a Sperry apparatus with a 50 cm high gravity‐fed pressure head (Sperry *et al*., 1988). We filtered deionized water (0.2 μm), mixed to a concentration of 100 mM KCl, and then degassed this perfusing solution using a membrane contactor (LiquiCell micro module; 3M, St Paul, MN, USA) connected to a vacuum pump following best practices (Cochard *et al*., 2013; Venturas *et al*., 2015). We then placed stem segments in perfusing solution overnight under vacuum, infiltrating them to their maximum conductivity (*K*_max_), which was verified with active xylem staining using 0.1% safranin solution after the *K*_max_ measurement was complete. We measured *K*_max_ using the same methods as for *K*_s_, corrected all conductivity measurements to sample dimensions, including length (Sperry *et al*., 1988), and calculated the per cent loss of conductivity:

$$\text{PLC}=100\left[1-\left(\frac{K_{s}}{K_{\max}}\right)\right]$$

Monitoring for survival or mortality occurred during the growing season, and we determined that a tree had survived drought if we observed new growth after rewatering. The last rewatering treatment occurred on 15 May 2017, after 125 d of drought. We analyzed the effect of PLC and *K*_s_ on mortality using logistic regression, a method appropriate for binary outcomes such as life or death (Menard, 2002), using the program R version 3.3.3 (R Core Team, 2013). Logistic models have been widely used for describing and predicting tree mortality and survival in response to environmental (Van Mantgem *et al*., 2009), tree growth (Cailleret *et al*., 2016) or ecophysiological conditions (Kursar *et al*., 2009; Anderegg *et al*., 2013; Bolte *et al*., 2016; Venturas *et al*., 2018). We assessed the fit of logistic regressions using a Nagelkerke *R* ^2^, a pseudo‐*R* ^2^ metric. Additionally, we conducted further active xylem staining on surviving trees to assess where in stems xylem remained functional after recovery.

We assessed foliar color using a Munsell plant tissue color book (Munsell Color, Grand Rapids, MI, USA) during three discrete stages of each tree's progression through the experiment. To measure foliar color, a representative fascicle of needles was removed from the upper third of each tree's canopy, and compared to the plant tissue color book for color matching (Tucker *et al*., 1991). Initial color was documented before water was withheld to initiate drought‐induced water stress, another measurement was taken at the rewatering treatment, and a final measurement was taken 60 d after rewatering. We transformed all color values from Munsell Hue/Value/Chroma into sRGB using the R package ‘aqp’ (Beaudette *et al*., 2013). We chose this color space because it is accurately represented on most digital devices, and allowed for comparison between trees that survived, and those that died, in color space. We used a Euclidean distance equation to calculate the Euclidean color distance between red (*R*), green (*G*) and blue (*B*) foliar color values at the initial (*i*, predrought) and rewatering (*f*, final) stages:

$$\sqrt{{\left(R_{f}-R_{i}\right)}^{2}+{\left(G_{f}-G_{i}\right)}^{2}+{\left(B_{f}-B_{i}\right)}^{2}}$$

We tested for differences in Euclidean color distance (from initial to rewatered canopy foliar color) between watered controls, trees that survived and those that died using ANOVA, with *post hoc* testing using Fisher's least significant difference (LSD) test, in the program R.

## Results

**Figure 1:**
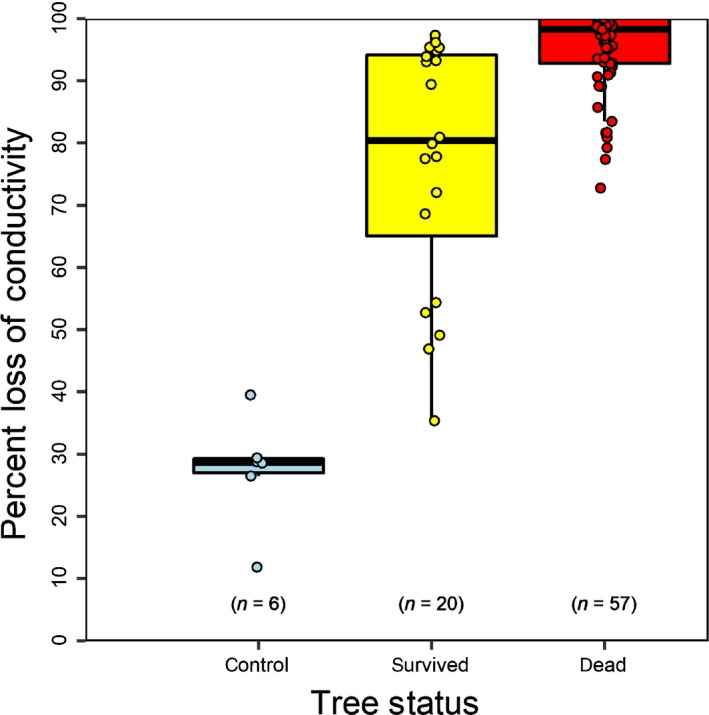
Native (directly measured) per cent loss of hydraulic conductivity (PLC) in *Pinus taeda* that died or survived drought, or were watered throughout the experiment (control). PLC values shown for drought trees were measured just before rewatering. Watered control PLC measurements were taken at the end of the experiment. All trees that died had a directly measured PLC exceeding 70. Central bars indicate median values, boxes represent the interquartile range (IQR) and whiskers extend to the maximum and minimum data value within 1.5 × IQR. Data points are jittered to reveal overlapping points. Median values are significantly different from each other for all three groups (Kruskal–Wallis test, *P* < 0.001).

Our drought followed by rewatering treatments at variable water potential targets in 77 saplings provided the intended wide variation in PLC at rewatering that ranged from 35.4 to 100.0 PLC (Table S1). After allowing at least 60 d for recovery following rewatering, we determined whether trees resumed growth (survived) or had complete canopy desiccation and browning (died). We found that 57 trees died and 20 survived. From measurements taken on the day drought stress was relieved, the mean (SD) PLC of trees that eventually recovered was 77.2 ± 19.7, while trees that would die averaged 94.5 ± 6.9 PLC (Fig. 1). All trees that died had a PLC > 72 at rewatering. Control trees that were watered weekly during the experiment (*n* = 6) had a mean PLC of 27.5 ± 9.7. Median values for the three groups (control, survived drought, died) were significantly different (Kruskal–Wallis test, *P* < 0.001, Fig. 1).

**Figure 2:**
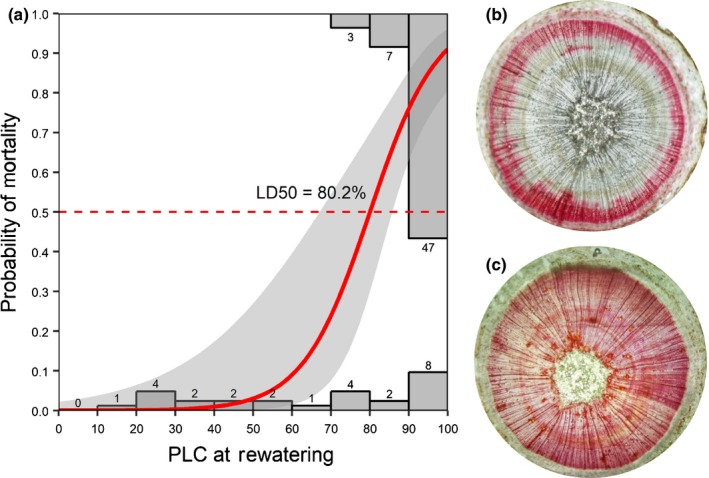
A logistic regression to determine the 50% lethal dose (LD50, dashed red line) of per cent loss of hydraulic conductivity (PLC) during drought, which was 80.2 (95% Wald confidence for probability of mortality had a minimum PLC = 67.1 and maximum PLC = 85.5 at LD50; panel a). Bars represent the proportion of all trees in each 10% PLC bin, scaled to the height of the *y*‐axis, with the count of trees per bin labeling each bar. The solid red line is the logistic regression fit, with shaded gray area representing a Wald 95% confidence interval for the logistic regression. The predicted lethal threshold for hydraulic failure, PLC = 60, was not contained in this interval at LD50 (dashed red line). The majority of trees which died (*n* = 47) did so with a PLC > 90. (b,c) Stem cross‐sections of *Pinus taeda* with functional xylem stained red using 0.1% safranin. (b) Stem of a tree at 83.4 PLC, past the hydraulic failure point of no return, which died following rewatering. (c) A well‐watered control tree, with all xylem functional.

Using logistic regression, we determined the lethal threshold for hydraulic failure, at which 50% of trees would die (LD50), to be 80.2 PLC (Nagelkerke *R* ^2^ = 0.53, Fig. 2). A Wald 95% confidence interval (CI) for our logistic regression predicting the mean probability of mortality contained a minimum of 67.1 PLC and a maximum of 85.5 PLC, but did not include PLC 60, the hypothesized PLC at 50% probability of mortality. Probabilities for mortality were 0.03 (95% CI: 0.00–0.27) for 50 PLC, 0.09 (95% CI: 0.01–0.39) for 60 PLC, 0.24 (95% CI: 0.08–0.54) for 70 PLC and 0.76 (95% CI: 0.63–0.86) for 90 PLC. The specific native conductivity of stems (*K*_s_) measured immediately before rewatering ranged from 0.37 kg m^−1^ MPa^−1^ s^−1^ to 0 (no flow), and using logistic regression we determined LD50 in *K*_s_ of 0.06 kg m^−1^ MPa^−1^ s^−1^ (Nagelkerke *R* ^2^ = 0.51, Fig. S2), which was an 83.8% loss of the maximum observed *K*_s_. We provide our fitted probability functions for mortality from PLC and *K*_s_, and the confidence intervals for both in Tables S1 and S2, respectively.

**Figure 3:**
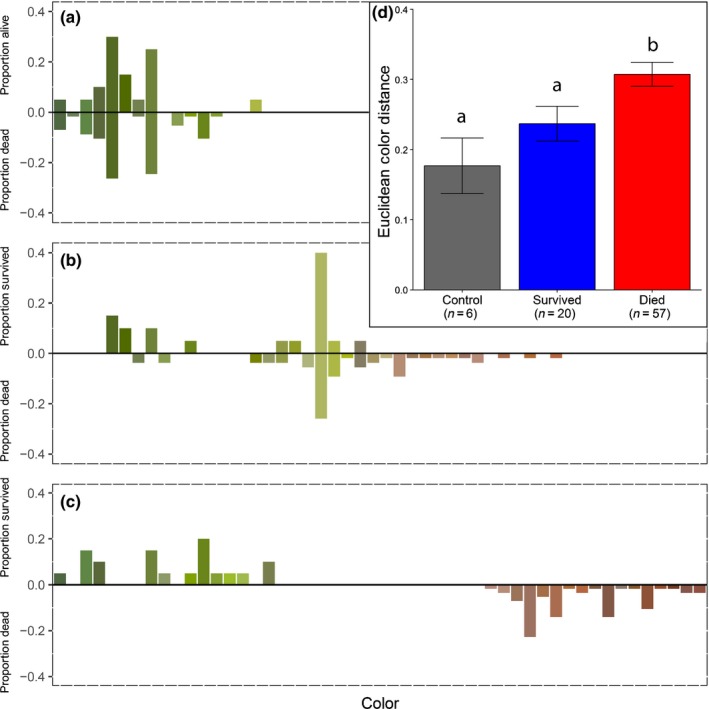
Observed canopy color of *Pinus taeda*, arranged from darkest green to deepest red‐brown. Bar height indicates the proportion of trees that survived (positive proportion) or of trees that died (negative proportion) at a given color. Bars are filled with the same color we observed in the tree foliage. (a) Canopy foliar color at the beginning of the experiment, before drought, and foliar color of all trees was a deep green. (b) Canopy foliar color at rewatering, with trees that survived tending toward green, while trees that died tending toward yellow/brown colors. (c) Canopy foliar color 60 d after rewatering, where trees that survived have returned nearer to initial foliar color positions and trees that died all experienced browning of foliage. Data in (a–c) are for trees exposed to drought that either survived or died; analogous data for the watered control trees are shown in Supporting Information Fig. S3. (d) The Euclidean color distance, a calculation of the change in color between predrought (a) and rewatering (b) for control trees (grey) and trees that experienced drought and survived (blue) or died (red). Trees that died during drought stress had a significantly greater Euclidean color distance than trees which survived (whether they experienced drought, or were a watered control; ANOVA, *F*_2,80_ = 4.737, *P* = 0.0114, *post‐hoc* Fisher's LSD test). Letters above bars in (d) indicate significant differences and error bars are ± SEM.

**Figure 4:**
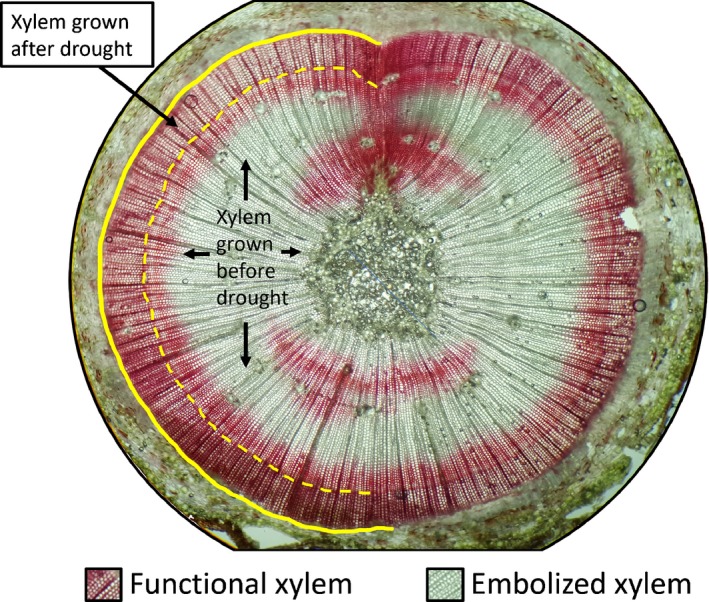
Cross‐section of a stem segment taken from an active xylem stain of a loblolly pine (*Pinus taeda*) tree which experienced extreme (96 PLC) hydraulic failure, yet recovered after rewatering. This cross‐section was taken from a stem segment excised 85 d after rewatering, after radial growth had occurred following relief of the drought. Active xylem is indicated by red (stained by 0.1% safranin solution), while embolized xylem is unstained. During the drought, the vascular cambium's location is indicated by a yellow dashed line, while the position of the vascular cambium at the time of cross‐sectioning is indicated by a solid yellow line. As can be seen, xylem remained functional close to the location of the vascular cambium at rewatering (indicated by a yellow dashed line) of this tree surviving extreme stress, and hydraulic function after 85 d of recovery has been restored via new radial growth. All markers appear only on the left half of the cross‐section, so that the right side may be inspected visually.

Euclidean color distance between initial canopy foliar color and canopy foliar color at rewatering was significantly higher for trees that died than for those that either survived drought or did not experience drought (ANOVA, *F*_2,80_ = 4.737, *P* = 0.0114, *post‐hoc* Fisher's LSD, Fig. 3d). The general trend for trees that died was a change from green to a more yellow or brown foliar color (Fig. 3a,b). Sixty days after rewatering, foliar color had returned to a deep green for trees that survived, and trees that died had complete foliar browning (Fig. 3c). Observations from active xylem stains of stems from recovered trees revealed that embolism remained in tissues which experienced the drought treatment, but that surviving trees had active xylem near the vascular cambium when drought was relieved, which remained functional during our stains (Fig. 4). Additionally, embolism from the drought remained in the interior of the stems when sections were made, 90 d after relief of drought (Fig. 4).

## Discussion

We found that the point of no return (LD50 at the population level) for directly measured PLC at rewatering was 80.2, indicating that some loblolly pine saplings were able to survive with < 20% (or even 10%) of their xylem functional at rewatering. Interpolating from our results, we would predict only 9% (95% CI: 1–39%) mortality at 60 PLC, much lower than the 50% mortality (LD50) we expected based on previous indirect estimates (Brodribb & Cochard, 2009; Choat *et al*., 2018). Observations of *Pinus halpensis* (Miller) under drought in the field noted that trees can survive > 80 PLC (Klein *et al*., 2012), but we believe our result is the first direct determination of LD50 for PLC to quantify the point of no return with measured individuals monitored for survival or mortality. Our experiment provides a hydraulic failure function for risk of mortality that can be directly input to vegetation models (e.g. TREES, CLM(ED(X)), SurEau; Mackay *et al*., 2015; McDowell *et al*., 2016; Martin‐StPaul *et al*., 2017; Johnson *et al*., 2018), although more work is needed to generalize these functions across life‐stages and species (see the third from last paragraph of the discussion). Our results enable predictions of mortality risk that can be based on either a threshold that represents the point of no return from hydraulic failure (LD50), rather than complete desiccation of the xylem (i.e. 100 PLC), or a continuous prediction of mortality risk across all possible PLC values.

It is remarkable that some pine trees were able to survive extreme levels (> 90 PLC) of hydraulic failure. In our study, trees were rewatered completely (to field capacity) on the day we measured the target PLC, and weekly thereafter. This rapid cessation of drought and switch to abundant soil moisture in our experiment could have enabled increased survival probability at very high PLC. Drought in the field is not typically relieved so immediately, completely and persistently, which could result in fewer trees surviving very high PLC under natural conditions. Our data suggest that as long as there was supply of water to the vascular cambium, and xylem tension was able to relax before complete (P100) hydraulic failure, there is a chance for survival. Our measurements of both hydraulic conductivity and active xylem staining after rewatering showed that surviving trees restored conductivity via radial growth (Fig. 4). Therefore, an ability to grow new xylem tissue following drought may be key to a tree's long‐term survival. Past studies estimated 50 PLC to be a likely threshold for life or death for gymnosperms (Brodribb & Cochard, 2009; Choat *et al*., 2018), yet no trees rewatered below 72 PLC died in our experiment. Additional species should be investigated to determine if the ability to survive such extreme hydraulic failure (e.g. > 90 PLC) is widespread for gymnosperm trees.

Characterizing mortality risk along a drought‐stress gradient provides a critical parameter for global vegetation modeling (Hartmann *et al*., 2018a). While our finding provides a point of no return (LD50), beyond which it is more likely trees will die than survive, we also characterize risk across the entire range of possible values for hydraulic failure. Indeed, our model predicts < 5% mortality until PLC > 55. At the other extreme, our model predicts 75% probability of mortality at 90 PLC (Fig. 2). Utilizing our continuous probability predictor of mortality risk, even at high levels of PLC some trees are predicted to survive – as is often observed in forests affected by drought‐induced tree mortality. Thus, we recommend utilization of continuous predictors of mortality risk over threshold‐like approaches only including binary outcomes of 100% life or death on opposing sides of the threshold (Hartmann *et al*., 2018a).

When investigating what kills trees under drought, the definition and quantification of death matter if we wish to truly determine the mechanism involved. In the present study, we have quantified the point of no return from drought stress – such that if the lethal stressor is relieved, there is still a failure to recover and death results. It is especially important to distinguish between physiological causes of death, assessed at the point of no return when trees begin dying, and noncausative symptoms or correlates that occur after this point and are evident at tree death (Adams *et al*., 2017; Hartmann *et al*., 2018a). Symptoms that have often been used to infer tree death include foliar browning and other visual indications of dieback (Adams *et al*., 2009; Anderegg *et al*., 2012), and cessation of respiration, assessed directly, or through tissue staining (Sevanto *et al*., 2014). It is possible that physiological variables measured when such criteria are met are those for a tree already long past the point of no return, which has been committed to die for some time. Physiological responses measured after the point of no return (e.g. at foliar color change or cessation of respiration) cannot have caused death, and may differ from responses measured at or before the point of no return. Future studies seeking to understand the physiology of tree death from drought should measure additional responses to drought stress (e.g. NSC, spectral reflectance, water content) at the point of return (if known, or if not, along a gradient of drought stress) to determine if responses precede, follow or are concurrent with the point of no return from hydraulic failure (Martínez‐Vilalta et al., 2019; Sapes et al., 2019).

Although we observed that foliar color in trees was significantly different at the time of rewatering, there was much overlap in canopy color between trees that survived and those that died (Fig. 3b,d). Foliar color was not wholly separated between these groups until our measurement at 60 d after rewatering (Fig. 3c), and thus was a better indicator of trees that are already dead, rather than those which are in the process of dying. Studies relying on foliar color to determine the timing of tree death have probably overshot the moment when trees passed the point of no return, and therefore measurements of physiological function at this point can confuse noncausative symptoms of having died for actual causes of mortality (Leuzinger *et al*., 2009). However, measurement of foliar color after tree death, rather than at the point of no return, has remote sensing applications for detecting recent tree mortality in evergreen forests, before changes become obscured by a green understory, provided a baseline color is available before drought (Hartmann *et al*., 2018a). Improved characterization of foliar color changes at additional spectra as hydraulic failure occurs during drought may improve the predictive power of foliar color in remote sensing of drought‐induced tree mortality, an approach which will require additional experimentation to measure across an array of wavelengths.

By directly measuring PLC across a water‐stress gradient ranging from mild to lethal in loblolly pine saplings, we observed that the lethal threshold was higher than our expectation of 60%. Our observation raises the following question: Do other species exhibit similarly high lethal thresholds for hydraulic failure? We hypothesize that this threshold may be relevant for conifer species, and that species differences in hydraulic vulnerability affect timing to reach the lethal threshold in PLC, rather than alter the threshold directly. Here it is important to distinguish between the lethal threshold in PLC, a normalized response for the loss of hydraulic function, which may correlate consistently with mortality risk across species, and the degree of tension in the xylem required to reach this threshold in a given tree, which is not normalized (Cochard *et al*., 2013). Vulnerability to embolism, quantified as the relationship between water potential and PLC, has been observed to vary by nearly an order of magnitude in gymnosperms, with water potential corresponding to 50 PLC spanning −1.5 MPa (*Podocarpus latifolius*) to −14.1 MPa (*Actinostrobus acuminatus*; Maherali *et al*., 2004). We also expect wide variation in traits which mediate the progression of water potential – and xylem embolism accumulation – during drought. Such traits include stomatal control, minimum conductance rates following stomatal closure, allocation and leaf area to root or sapwood area ratios, root water uptake and hydraulic isolation from the soil (Choat *et al*., 2018; Blackman *et al*., 2019; Hammond & Adams, 2019). If, as multiple lines of recent research have suggested, a major evolutionary pressure exists to keep woody tissues from experiencing damaging levels of hydraulic impairment (PLC; e.g. Sperry *et al*., 2016; Wolf *et al*., 2016; Martin‐StPaul *et al*., 2017; Anderegg *et al*., 2018), and if embolism repair is relatively rare (Brodersen & McElrone, 2013; Choat *et al*., 2015), then lethal thresholds in PLC may be fairly consistent across species. However, plant traits and species differences will influence how and when species reach this threshold. Experiments are needed to test whether lethal thresholds in PLC vary with species, phylogeny, anatomical factors such as lumen area and xylem cell wall thickness, environmental factors, including temperature and vapor pressure deficit, and nonstructural carbohydrate status (Trifilò *et al*., 2017; Martínez‐Vilalta *et al*., 2019).

Given that experimental data similar in nature are lacking for mature trees, one important consideration is how these results apply to larger trees under field conditions. While it is true that earlier developmental stages (e.g. seedlings, saplings) frequently experience higher rates of background mortality in the field (Van Mantgem *et al*., 2009), there are at least two contributing factors that should be considered. First, it is important to recognize that earlier developmental stages utilize considerably less environmental space (above‐ and below‐ground) and store less water and carbon than their mature counterparts and are thus more strongly affected by atmospheric demand and soil moisture during drought (Hartmann *et al*., 2018b). Second, anatomical changes across the ontogeny of a tree could affect vulnerability to drought. Such changes may lead to a difference in the level of tension in the xylem (water potential) required to reach a lethal level of hydraulic failure (such as our observed 80 PLC). Both of these factors could contribute to an earlier death of seedlings and saplings during drought relative to larger and more mature trees, even if the LD50 for PLC was unchanged with ontogeny. Notably, P50 in mature *Pinus taeda* trees (quantified as water potential inducing 50 PLC) was found to be −3.13 MPa (Maherali *et al*., 2006), which is within the 95% confidence interval of P50 for the vulnerability curve we measured in our sapling population of the same species (−3.22 MPa, Fig. S1). Acknowledging that our experimental approach would be challenging for mature trees in a forest, we propose that the best way to validate extrapolations of mortality risk from sapling experiments is to use this type of experimental data to explain past mortality in the field before attempting to predict mortality under future climate scenarios (Anderegg *et al*., 2015).

Our direct measurement of a lethal PLC threshold of loblolly pine near 80% demonstrates that the point of no return was higher than expected for a gymnosperm species, and that some trees can survive near complete hydraulic failure. In experimental determinations of lethal hydraulic thresholds in angiosperms, previous studies indicate that trees can survive 88 PLC, but these studies inferred PLC from measurements of water potential using vulnerability curves, rather than direct measurement (Urli *et al*., 2013; Li *et al*., 2015), although direct measurements of PLC in two angiosperm species support a high threshold (Barigah *et al*., 2013). In combination, these results and our finding of a relatively high LD50 for PLC in loblolly pine raise questions of how lethal thresholds vary among tree species, populations and ontogeny. Continued experimentation will be necessary to assess this, but we hypothesize that differences among trees (both within and between species) in vulnerability to drought‐induced mortality may be primarily caused by variable embolism resistance, and variable rates of declines in water potential, more than differences in the degree of hydraulic failure (PLC) that is lethal. Overall, our results quantify tree mortality risk from hydraulic failure, a parameter critical for process‐based models of forest response to climate change.

## Author contributions

WMH and HDA conceived the study, assisted by REW. WMH, KY and LAW collected data during the study. WMH and HDA performed the analysis. WMH, WRLA, HDA and LAW interpreted the results, and WMH wrote the manuscript, with substantial assistance from the other authors.

## Supporting information

Please note: Wiley Blackwell are not responsible for the content or functionality of any Supporting Information supplied by the authors. Any queries (other than missing material) should be directed to the *New Phytologist* Central Office.

**Fig. S1** Vulnerability curve of *Pinus taeda*, loblolly pine, showing the relationship between per cent loss of conductivity and water potential.
**Fig. S2** Logistic regression model of mortality risk as a function of specific conductivity (*K*_s_).
**Fig. S3** Foliar color of watered control trees at three phases of the experiment: predrought, end of rewatering and recovery.

**Table S1** Logistic regression model predictions for the probability of mortality dependent on per cent loss of conductivity (PLC).

**Table S2** Logistic regression model predictions for the probability of mortality dependent on specific conductivity (*K*_s_).
