# Supplementary material for: Dead or dying? Quantifying the point of no return from hydraulic failure in drought‐induced tree mortality
Source: New Phytol. 2019 Jul 8;223(4):1834–43. doi: 10.1111/nph.15922 (PMC6771894; doi:10.1111/nph.15922)
Supplement: Supplementary file 1 — Fig. S1 Vulnerability curve of Pinus taeda, loblolly pine, showing the relationship between per cent loss of conductivity and water potential. Fig. S2 Logistic regression model of mortality risk as a function of specific conductivity (K s). Fig. S3 Foliar color of watered control trees at three phases of the experiment: predrought, end of rewatering and recovery. [file NPH-223-1834-s001.pdf]

Supplementary Information for

Dead or dying? Quantifying the point of no return from hydraulic failure in drought-induced tree mortality.

Hammond, William M., Yu, Kailiang.L., Wilson, Luke A.,3, Will, Rodney E., Anderegg, William R.L., and Adams, Henry D.

Article Accepted: 05 May 2019
Corresponding Author: William M. Hammond
Email: william.hammond@okstate.edu
Phone: 405.471.7203

This PDF file includes:

Figs. S1 to S3
Captions for data files Table_S1.csv and Table_S2.csv

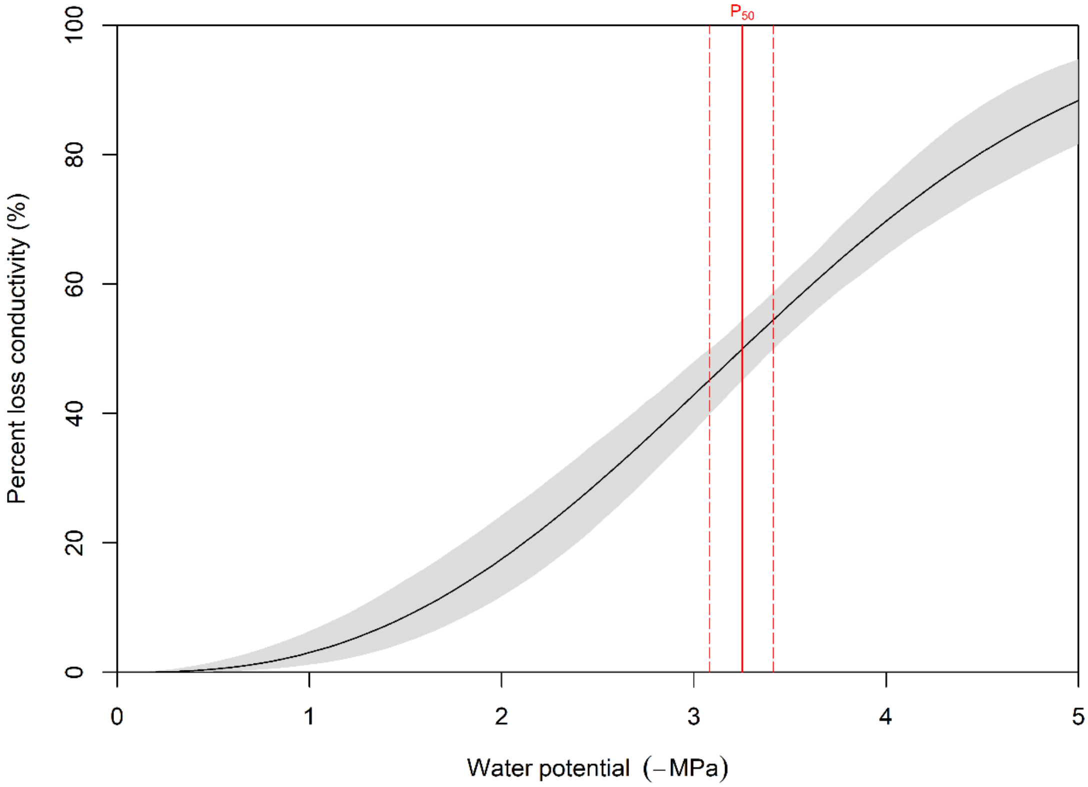

Figures S1. Vulnerability curve of *Pinus taeda*, loblolly pine, generated with samples from six saplings of the experimental population, using centrifugation to induce embolism. The water potential at 50 PLC (P_{50}), -3.22 MPa, is shown with a solid red line, and the 95% confidence interval for P50 (-3.05 to -3.37 MPa) is shown with dashed red lines. We fitted the curve using R package 'fitplc' (Duursma & Choat, 2017).

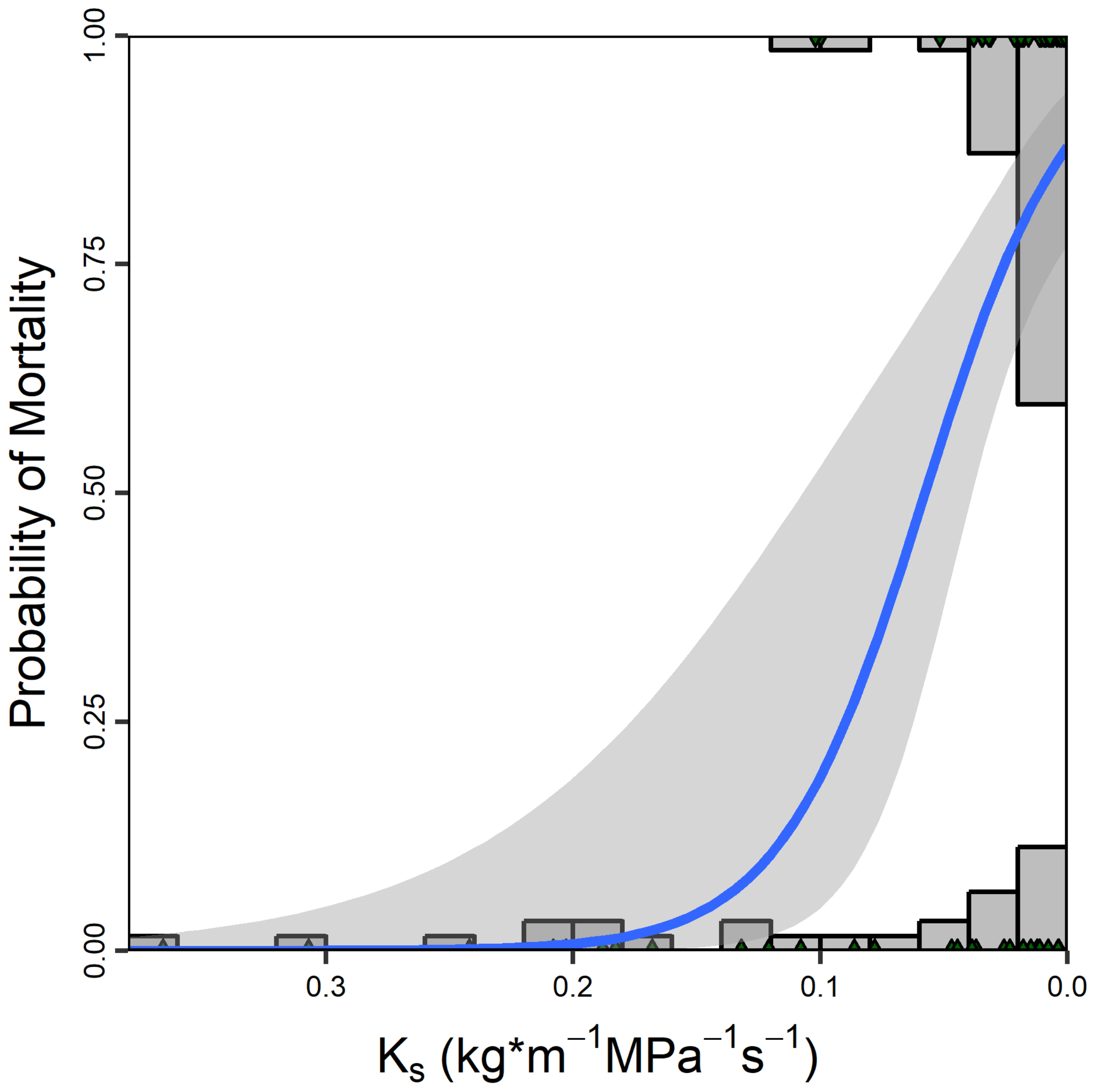

Figure S2. A logistic regression to determine the 50% lethal dose (LD50) of native conductivity (K_s) during drought, which was 0.06 (95% Wald Confidence for probability of mortality had a minimum K_s = .04 and maximum K_s = 0.11 at LD50). Bars represent proportion of all trees in 20 bins across the observed range of K_s, scaled to the height of Y-axis. Solid blue line is the logistic regression fit, with shaded grey area representing a Wald 95% confidence interval for the logistic regression.

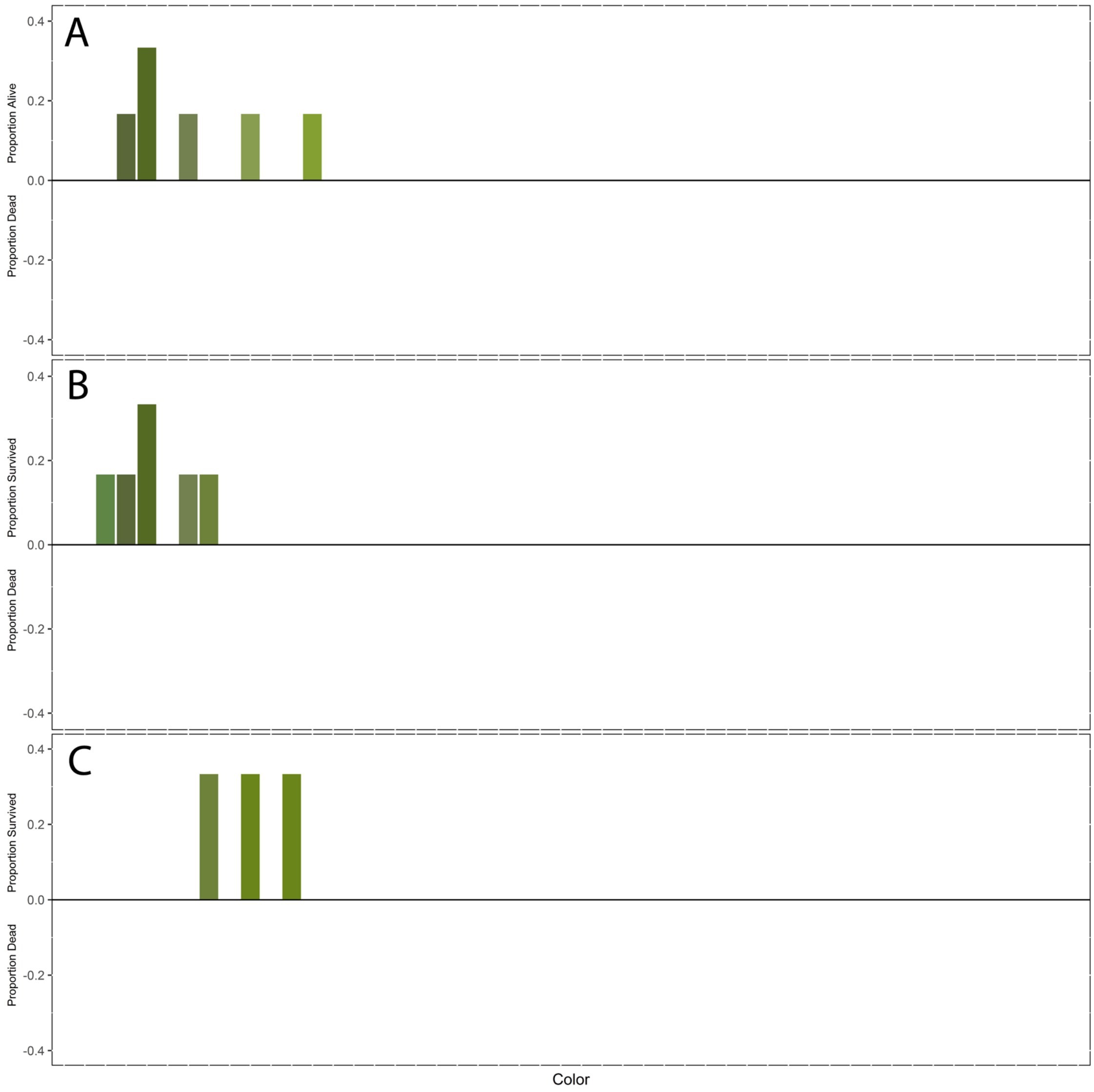

Figure S3. This figure is a companion to **Figure 3** in the main text—containing data for only the six watered control trees (which are not included in Figure 3). The x-axis remains the same as Fig. 3, observed canopy color, arranged from darkest green to deepest red-brown. Bar height indicates the proportion of trees that survived (positive proportions) or the trees that died (negative proportions) at a given color. Bars are filled with the observed foliar color recorded from a representative sample. Panel A shows canopy foliar color at the beginning of the experiment, before drought, and foliar color of all trees was a deep green. Panel B shows canopy foliar color at the end of the re-watering period. Panel C shows canopy foliar color 60 days after re-watering of the last droughted tree in the experiment. Canopy color was consistently green,

without yellowing or browning of the canopy during the experimental drought and recovery periods.

Table S1. Logistic regression model predictions for probability of mortality given percent loss of conductivity values (PLC) ranging from 0 to 100, by 0.1. Model fit is provided, along with lower (lwr) and upper (upr) 95% Wald CI's for probability of mortality. **(see separate .csv file)**

Table S2. Logistic regression model predictions for probability of mortality given specific conductivity values (K_s) ranging from 0 to 0.366 (the range of observed values during this experiment), by 0.001. Model fit is provided, along with lower (lwr) and upper (upr) 95% Wald CI's for probability of mortality. **(see separate .csv file)**

References:

Duursma RA, Choat B. 2017. fitplc: an R package to fit hydraulic vulnerability curves. *Journal of Plant Hydraulics* **4,** e002 https://doi.org/10.20870/jph.2017.e002.
